# Preliminary Review of Spine Tumor Radiologic, Intra-Operative and Histopathology Findings, Addis Ababa, Ethiopia

**DOI:** 10.4314/ejhs.v32i1.7S

**Published:** 2022-10

**Authors:** Mahder Tewodros Bezu, Amal Saleh Nour, Tesfaye Gizaw Tefera, Kibruyisfaw Zewdie Shumbash, Mersha Abebe Woldemariam

**Affiliations:** 1 Wolaita Sodo University, Ethiopia; 2 Department of Radiology, College of Health Sciences Addis Ababa University, Ethiopia; 3 Department of Surgery College of Health Sciences Addis Ababa University, Ethiopia

**Keywords:** Agreement, CT, Histopathology, MRI, Spine tumors

## Abstract

**Background:**

Spinal tumors constitute 10–32% of all primary central nervous system tumors. Accurate radiologic and histopathology diagnosis is crucial in the management and prognosis. The aim of the study was to describe the imaging patterns and to determine the agreement of imaging pattern of spinal tumors with intra-operative and histopathology findings.

**Methods:**

A retrospective cross-sectional study of 47 patients with spinal tumor done from May 2018 to October 2020. Medical records were reviewed for clinical data, history, physical examination, magnetic resonance imaging (MRI), intraoperative findings and histopathology reports. The agreement between imaging, intraoperative finding and histopathology diagnosis was analyzed.

**Results:**

Intradural extramedullary tumors constituted 37 (78%) cases followed by six (12.8 %) extradural tumors and four (9.2%) intramedullary tumors. Schwannoma accounted for 13 (27.7%) cases followed by meningioma, 12 (25.5%) cases. Twenty-seven (57.4%) cases were thoracic level and cervical level were nine (19.1%) cases. Twelve (25.5%) cases did not have a definite intraoperative diagnosis. Imaging and intraoperative diagnosis was in agreement in 21 (44.6 %) cases and disagreed in 14 (29.7%) cases. For the imaging diagnosis and histopathology, 29 (61.7%) were in agreement and 18 (38.3 %) were in disagreement.

**Conclusion:**

In conclusion, the commonest site to be involved was the thoracic spine and schwannoma was the commonest tumor. The low agreement between imaging and histopathology could have been improved by optimizing the imaging reports and techniques.

## Introduction

Spinal cord tumors constitute 10–32% of all primary central nervous system tumors and are usually classified based on their location (1,2). Lesions which are located outside the dura are termed extradural and lesions located within the dura are termed intradural. Intradural tumors are further categorized into two based on the involvement of the spinal cord, intramedullary if the lesion involves substance of the spinal cord and extramedullary if the lesion doesn't involve the spinal cord but located within the dura (1). Spinal tumors are predominantly located in the thoracic region followed by cervical region with the lumbar region being the least involved (1,3).

Intramedullary tumors accounts for 10–15% of spinal tumors and most are malignant (1,4). 80% of intramedullary tumors are made of gliomas which are further divided into astrocytoma and ependymoma which have about the same incidence (5).

Astrocytoma is more common in adults and commonly found in the thoracic region whereas ependymoma is more common in children and more common in the cervical region(1,3). Non-glial intramedullary tumors are less common and consists of hemangioblastomas, paragangliomas, metastases, lymphoma, and primitive neuroectodermal tumors (PNETs) (6).

Extra medullary tumors account for 70–90% of spinal tumors and mainly consist of schwannomas which is commonly located in the cervical and lumbar region followed by meningioma which is commonly located in the thoracic region (1).

Extradural tumors accounts for 25% of spinal tumors and mainly includes meningioma and metastatic disease (1). Metastatic diseases are rare and can occur as a result of hematogenous spread or direct extension from the leptomeninges. Primary extradural tumors are rare and the most frequent tumors are neurinoma followed by meningioma and lipomas (4).

Spinal tumor occurs predominately in the third decade and there is an equal occurrence in male and females except meningioma which is more frequent in women. These tumors manifest with a spectrum of sign and symptoms depending on the level and plane of the lesions (5). Patients usually present with neurological dysfunctions including motor, sensory, sphincter function and balance. Clinical data like patient age, symptoms, and history and laboratory findings are helpful in making radiologic diagnosis (7).

There are many radiological modalities available for spinal pathology diagnosis. These include myelography, spinal arteriography, CT and MRI. Conventional radiological views are currently considered as traditional since they can't assess extent of the lesion (7). CT and MRI are used for making early diagnosis of spinal tumors, furthermore, MRI is used as a primary imaging diagnostic modality and choice of preoperative assessment (5). MRI is also the best diagnostic modality for predicting neurological prognosis (8).

The MRI signal intensity and enhancement pattern of lesions help to give a differential diagnosis. Lesions with pseudo capsule and marked contrast enhancement were considered as ependymoma whereas lesions with eccentric location and patchy contrast enhancement are considered as astrocytoma. Hemangioblastomas was considered in a cystic lesion with enhancing nodule (5).

Studies have shown a 75% agreement between radiological and histopathology (HPE) as well as preoperative and HPE correlation. In the lumbar level, radiological and HPE correlation, 40% of them are in complete agreement and in preoperative and HPE correlation, 60% are in a complete agreement (5).

For intramedullary tumors, MRI aspects were correlated with tumor pathology in 70% of cases by using MRI characteristics (7). Radiologists play an important role in evaluating the extent of a lesion as well as topography and suggest a specific diagnosis, this in turn guides neurosurgeons in performing successful surgical interventions (9).

The aim of this study was to describe the imaging patterns and assess the agreement between the radiological, intra-operative and histopathology of spinal tumor.

## Materials and Methods

A retrospective institutional based cross sectional study design was used to assess the clinical and imaging patterns of spinal tumors. The agreement between imaging, intraoperative findings and histopathology was also assessed. The study was conducted at Tikur Anbessa Specialized Hospital (TASH), and Zewditu Memorial Hospital (ZMH) from May 2018 to October 2020.

The source population were all patients with spinal tumors who were referred to neurosurgery during the study period. The study population were all patients who have cross-sectional imaging, intraoperative findings and pathology results. Previously operated patients, as well as patients with incomplete medical records were excluded from the study. This study was conducted by reviewing the medical records of patients with spinal tumors who came to the department of neurosurgery.

The medical records of 81 patients were reviewed, 34 had incomplete data and were excluded leaving 47 patients. The patient's demography, history, physical examination, CT, MRI reports, intraoperative findings and histopathology reports were reviewed and filled in a structured questionnaire.

**Statistical analysis**: Data entering, coding and clearing for the quantitative data was performed using Microsoft excel and the analysis was performed with SPSS version 25. The sociodemographic, clinical characteristics and intraoperative findings were computed by using simple descriptive statistics (mean, percentage, frequencies). Means and ranges were calculated from continuous variables. The agreement between imaging, intraoperative and histopathology diagnosis was analyzed using cross tabulation.

Agreement is present when the imaging diagnosis or the first imaging diagnosis in the list of differential diagnosis is similar with the intraoperative or histopathology diagnosis.

**Ethical consideration**: Ethical clearance was obtained from the ethics committee of the department of radiology before the commencement of the study.

## Results

In our study, there was a total of 47 patients with spinal tumors, 29 (61.7%) male and 18 (38.3%) female with a male-to-female ratio of 1:1.6. In nine (19.1%) patients, there was no mention of the primary presenting symptom. In 38 patients (80.9%), the primary clinical presentation was stated and lower extremity weakness was seen in nine (19.1%) patients followed by lower extremity pain in five (10.6 %) patients.

Based on the imaging findings, intradural extra medullary tumors constituted 37 cases (78.7%) of the spinal tumors followed by extradural tumors which constituted six (12.8%) cases and intramedullary tumors which constituted four (8.5%) cases. Twenty-seven (57.4 %) cases involved the thoracic level, nine (19.1 %) cases involved the cervical level and five (10.6 %) cases were located in the lumbar level, three (2.1%) cases were located in the lumbo-sacral level and one case was located in the sacral region.

**Figure 1:**
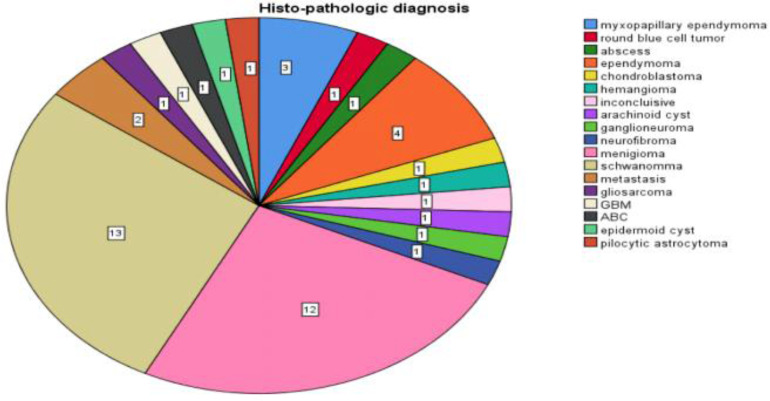
Type of spinal tumors distribution in patients evaluated during the period of February 2017–January 2020.

Schwannoma constituted 13 (27.7 %) cases of spinal tumor and nine (69%) of them are male patients. Meningioma was diagnosed in 12 cases (25.5 %) and eight (66.7%) were female. Ependymoma and metastasis represented four (8.5%) cases and two (4.3%) cases respectively. There was one case each of the remaining tumors in 16 cases (34%) as shown in Figure 1.

**Figure 2:**
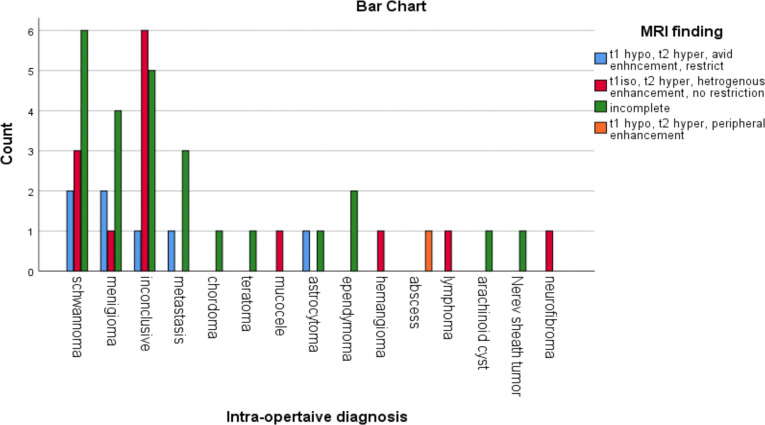
MRI characteristics of spinal tumors in patients evaluated during the period of February 2017–January 2020.

The imaging patterns for meningioma was not described in seven (58.3%) cases out of the 12 cases. In the remaining five (38.5%) cases, T1 hypo intense, T2 hyper intense lesion with avid post contrast enhancement with restriction was described in three (30.8%) patients (Figure 2).

**Figure 3:**
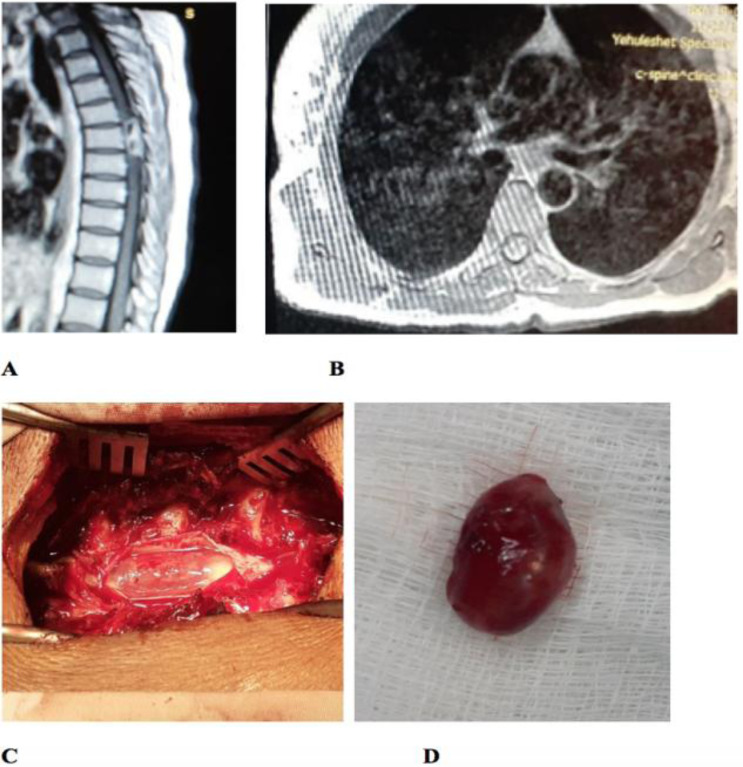
Post-contrast T1 image showed thoracic intraspinal heterogeneously enhancing mass diagnosed as schwannoma (A, B) Intraoperative photo showing the exposed mass and the removed mass (C, D).

The imaging patterns for schwannomas were not completely described in eight (61.5%) out of the 13 cases. In the remaining five (38.5%) cases with a complete imaging pattern description, the imaging pattern was T1 isointense, T2 hyper intense lesion with heterogeneous enhancement and no restriction in four (30.8 %) patients (Figure 3).

Out of the 47 cases, bony involvement was not mentioned on the imaging report for 30 of the cases (63.8%). No bony changes were seen in 10 (58.8 %) cases, lytic lesions were identified in four (23.5%) cases. The lesion was sclerotic in one (5.9 %) case and mixed lesions were seen in two (11.8%) cases.

There was no mention on imaging of the presence or absence of syrinx in 14 cases (29.8%). From those that mentioned syrinx in their reports, there was no syrinx seen in 28 cases (59.6%) and syrinx was present in five (17.8%) cases.

Twelve of the cases (25.5 %) did not have a definite intra-operative diagnosis while 35(74.5%) had a diagnosis. For those that had intra-operative diagnosis, there was agreement between imaging and intraoperative diagnosis in 21 (60%) with disagreement in 14 cases (40 %). The agreement between imaging diagnosis and histopathologic diagnosis was 29 (61.7%) and disagreement in 18 (38.3 %). The agreement between intraoperative diagnosis and histopathologic diagnosis was in 20 (57.1 %) cases. In 15 (42.9 %) cases results showed disagreement.

## Discussion

Among 47 spinal cord tumors, the mean age was 35.9 years and the majority of cases was seen in males in 29 (61.7%). The commonest tumors were intradural extra medullary constituting 37 cases (78.7%) of the spinal tumors followed by extradural tumors with six (12.8%) cases and intramedullary tumors with four (8.5%) cases, which correlates well with other studies done in India (1) 5). The commonest pathology were schwannomas in 13 (27.7%) cases followed by meningioma constituting 12 (25.5%) cases which correlated with other studies which showed schwannoma 33% and 35% meningioma in up to 28 %(1, 4) .

The commonest level to be involved was thoracic spine in our study which is in disagreement with a study done showing lumbar to be frequently involved site (8). On imaging patterns, the MRI characteristics of the spine lesions was incomplete in more than half of the schwannoma and meningioma cases. Although most was incomplete, from the reports that mentioned the pattern, the commonest imaging pattern was T1 isointense, T2 hyperintense with heterogeneous enhancement and no restriction for schwannomas. For the imaging pattern of meningioma, the commonest imaging pattern was T1 hypo intense, T2 hyper intense lesion with avid post contrast enhancement and with restriction which is consistent with other studies (8).

Important imaging features that should be mentioned in reports were absent in a significant number of cases such as adjacent bone involvement in 30 cases (63.8%) or whether syrinx is present or not in14 cases (29.8%). The presence or absence of dural tail was not mentioned in our study which is an important imaging characteristic to differentiate between meningiomas and other tumors. Although cap sign is a helpful differentiating finding on imaging for schwannomas, no reports mentioned it's presence or absence. These are crucial in narrowing down the differential diagnosis on MRI which in turn impacts the surgical approach and operable decisions.

In our study, 12 (25.5 %) of the cases did not have a definite intra-operative diagnosis. For the remaining cases, there was agreement in 21 (60%) cases and disagreement in 14 (40 %) cases between intra-operative diagnosis and imaging diagnosis. We found no study to compare these finding with.

In agreement between imaging with that of histopathologic diagnosis, 29 (61.7 %) was in agreement and 18 (38.3%) was in disagreement, this finding correlated with a study done in India that showed 60% agreement (5). Although there are studies with similar degree of agreement, this number could have been higher if advanced imaging is utilized such as functional MRI. The agreement between intraoperative and that of histopathologic diagnosis was 20 (57.1%) and the disagreement was 15 (42.9 %). This number is lower than other studies which showed a better agreement of 68% (5).

It is the recommendation of this study to create awareness on the need to have complete MRI reports as this is crucial to make a proper pre-operative diagnosis. Although histopathology is the gold standard, in the absence of advanced imaging in resource limited setup, it is essential to put the most likely diagnosis in the list of differentials of tumors.

The limitation of this study is that the radiologic images could not be reviewed and we had to rely on the reports which did not mention complete imaging patterns. A prospective study could have alleviated some of the incomplete descriptions.

In conclusion, the commonest site to be involved was the thoracic spine and schwannoma was the commonest tumor. The low agreement between imaging and histopathology could have been improved by optimizing the imaging reports and techniques.
